# Cognitive Dispersion Predicts Grip Strength Trajectories in Men but not Women in a Sample of the Oldest Old Without Dementia

**DOI:** 10.1093/geroni/igab025

**Published:** 2021-07-28

**Authors:** Tamlyn Watermeyer, Fernando Massa, Jantje Goerdten, Lucy Stirland, Boo Johansson, Graciela Muniz-Terrera

**Affiliations:** 1 Edinburgh Dementia Prevention, Centre for Clinical Brain Sciences, University of Edinburgh, Edinburgh, UK; 2 Department of Psychology, Faculty of Health & Life Sciences, Northumbria University, Newcastle, UK; 3 Instituto de Estadistica, Universidad de la Republica del Uruguay, Montevideo, Uruguay; 4 Department of Epidemiological Methods and Etiological Research, Leibniz Institute for Prevention Research and Epidemiology—BIPS, Bremen, Germany; 5 Department of Psychology & Centre for Ageing and Health (AgeCap), University of Gothenburg, Goethenburg, Sweden

**Keywords:** Cognition, Epidemiology, Frailty, Longevity, Psychometrics

## Abstract

**Background and Objectives:**

Grip strength is a reliable marker of biological vitality and it typically demonstrates an expected decline in older adults. According to the common-cause hypothesis, there is also a significant association between cognitive and physical function in older adults. Some specific cognitive functions have been shown to be associated with grip strength trajectories with most research solely focused on cutoff points or mean cognitive performance. In the present study, we examine whether a measure of cognitive dispersion might be more informative. We therefore used an index that quantifies dispersion in cognitive scores across multiple cognitive tests, shown to be associated with detrimental outcomes in older adults.

**Research Design and Methods:**

Using repeated grip strength measures from men and women aged 80 and older, free of dementia in the OCTO-Twin study, we estimated aging-related grip strength trajectories. We examined the association of cognitive dispersion and mean cognitive function with grip strength level and aging-related rate of change, accounting for known risk factors.

**Results:**

Cognitive dispersion was associated with grip strength trajectories in men and the association varied by mean cognitive performance, whereas we found no association in women.

**Discussion and Implications:**

Our results provide evidence of a sex-specific vitality association between cognitive dispersion and aging-related trajectories of grip strength. Our results support the call for integration of sex and gender in health promotion and intervention research.

**Translational Significance:** Variability or inconsistency in cognitive performance is proposed as a sensitive measure of decline in cerebral integrity. Our findings suggest that this measure is associated with a decline in hand grip strength, a measure of vitality and physical status, in octogenarian men but not women, even though there were no overall differences in variability or mean cognitive performance between genders. These findings suggest that different factors contribute to vitality or physical status in men and women in the oldest old and highlight the need for targeted strategies to support health promotion and intervention between the sexes in later life.

Increases in life expectancy are well documented (Prince et al., 2013). As an individual’s life span is extended, the preservation of physical and cognitive health becomes critical for functional integrity, well-being, and quality of life (Paggi et al., 2016; Samy et al., 2020). Grip strength is often shown to be an indicator of physical strength and vitality and thereby a simple and overall measure of the integrity of the central nervous system that signals changes in underlying aging processes (Salthouse et al., 1998).

Grip strength is repeatedly found to be a reliable indicator of poor health in older adults. For instance, a study of the over half a million participants of the UK Biobank reported an association of grip strength with cardiovascular, respiratory, and cancer outcomes as well as with all-cause mortality (Celis-Morales et al., 2018). Furthermore, grip strength from midlife onward has also been shown to be associated with other detrimental events such as falls (Salthouse et al., 1998; Yang et al., 2018), hip fractures (Denk et al., 2018), and depression (Ashdown-Franks et al., 2019). As a result of these findings, grip strength has been suggested as a “vital sign” in middle-aged and older adults (Bohannon, 2008; Carson, 2020); a reliable biomarker for vitality and aging (Zammit et al., 2019). Grip strength is related to muscle mass, and as men have more muscle mass than women, the differences are reflected in grip strength too. Furthermore, grip strength differences between men and women can be attributed to differences in behavior that result in men engaging in more manual and physical labor than women over the life course (Vianna et al., 2007). However, the evidence about differences in the rate of change in grip strength between men and women is mixed (Cooper et al., 2011), with some reporting that men decline faster than women (Frederiksen et al., 2006), while others report the opposite (Rantanen et al., 1997).

The association between grip strength and cognition is even less understood. The common-cause hypothesis suggests a relationship between the cognitive and sensory domains and that aging-related changes in both reflect brain aging (Baltes & Lindenberger, 1997). Age-related loss of sensory receptors and neurons tend to produce a slowing of perceptual processing and less effective and slower encoding of new information (Anstey et al., 2001). This may directly lead to a slowing of cognitive processing and hence result in compromised cognitive performance. Most studies of the association between cognitive function and grip strength have so far only focused on single cognitive domains. For example, cross-sectional studies reviewed in 2018 by Kobayashi-Cuya et al. (2018) identified 22 publications that examined associations between cognition and hand grip strength in community-dwelling older adults. The majority of these studies only used the Mini-Mental State Examination screening device (Folstein et al., 1975). There is currently a lack of clear evidence of the association of grip strength with more detailed measures of various cognitive domains. Importantly, studies that have evaluated the association of multiple measures have typically considered them independently and the findings are so far mixed (Kobayashi-Cuya et al., 2018).

Longitudinally, results about the role of cognition on grip strength trajectories are scarce. As with cross-sectional studies, most longitudinal investigations have focused only on measures of global cognition (Kim et al., 2019) or single tests (Viscogliosi et al., 2017). Furthermore, studies on cognitive domains have typically only examined their independent association with changes in grip strength (Zammit et al., 2019), ignoring functioning in other cognitive domains, or looked into the correlation between changes in cognitive domains and grip strength, either in the context of aging-related decline (Zammit et al., 2021) or terminal decline (Björk et al., 2016).

Traditionally, cognitive performance is indexed by comparing an individual’s performance against established cutoff criteria or a reference group mean. However, the study of an individual’s inconsistency in performance across tasks or domains is gaining momentum in the neuropsychological and clinical literature (Costa et al., 2019). While performance inconsistency can be quantified in several ways, cognitive dispersion (CD), in which the standard deviation in an individual’s performance is measured across a set of cognitive measures or domains, is nowadays more commonly applied (Stawski et al., 2019). Dispersion across cognitive performance scores may signal subtle breakdowns in cognitive ability (Costa et al., 2019) and is therefore more likely to provide a more sensitive measure of early decline, relative to mean performance and/or single indicators of cognition (Cherbuin et al., 2010; Tales et al., 2012). It may also reflect subtle changes in cognition that can be detected before conventional neuropsychological thresholds for cognitive impairment are met (Koscik et al., 2016; Roalf et al., 2016). In this respect, CD can be conceptualized as a single representation of function across multiple domains subserved by several cortical regions and neural networks. Consequently, it may be considered a signature of decline in cerebral integrity (Holtzer et al., 2020). In fact, its association with indicators of brain pathology is well documented in studies that show associations between dispersion and smaller corpus callosum volumes (Anstey et al., 2007), global and regional white matter degeneration (Jackson et al., 2012), and faster entorhinal and hippocampal atrophy rates (Bangen et al., 2019). In fact, in a previous study using data from the OCTO-Twin Study of the oldest old, the study of Swedish twins aged 80 and older at study entry that we also study here, we showed that CD was associated with increased risk of dementia (Watermeyer et al., 2020a).

In the context of this evidence, the investigation of an association between CD and grip strength deterioration represents an opportunity to further test the common-cause hypothesis and examine CD as a novel marker of decline in physical function, such as muscular strength.

To the best of our knowledge, no research has examined whether CD is associated with grip strength trajectories in older adults, which inspired our investigation about whether CD is associated with trajectories of grip strength in the OCTO-Twin Study of the oldest old. We hypothesize that (a) CD will be negatively associated with grip strength level and (b) CD will be positively associated with the rate of change (i.e., decline) in men and women.

## Method

### OCTO-Twin Sample

The sample used in these analyses was drawn from the comprehensive longitudinal Origins of Variance in the Old-Old: Octogenarian Twins (known as the OCTO-Twin Study (McClearn et al., 1997), based on the oldest cohort of the Swedish Twin Registry. The sample includes 702 participants, with 351 complete twin pairs born in 1913 and earlier, who were or became 80 years of age during the first wave of data collection (1991–1993). Participants were tested at home or in institutional settings, by medical research nurses who were specially trained and regularly supervised. Participants were reassessed every 2 years with a total of 8 years of follow-up. The average rate of attrition from one testing wave to the next was 20% (10% per year), primarily due to death. Due to the secondary data analysis nature of this study, power calculations were not conducted for this article. Full details of the study population characteristics have been published previously (Cederlof & Lorich, 1978; McClearn et al., 1997; Pedersen et al., 2002).

In order to identify cases of dementia, a multidisciplinary team consisting of a physician and two neuropsychologists reviewed cognitive test results and medical records, including reported medical history, medication use, and self-reported information about diseases. Independent classification performed by another physician produced only marginal amendment (for more information on this procedure see the work of Johansson & Zarit, 1995). Dementia was diagnosed by consensus according to the third edition of the Diagnostic and Statistical Manual of Mental Disorders.

#### Ethics

The OCTO-Twin Study received approval from the Ethics Committee at the Karolinska Institute in Stockholm and from the Swedish Data Inspection Authority. Informed written consent was obtained from all participants or their relatives or carer where the capacity to consent was questionable, for example, due to severe cognitive impairment or dementia.

#### Grip strength

A Martin Vigorimeter (Elmed Inc., Addison, IL; medium size bulb) to measure maximum force in pounds per square inch was used. Participants performed the task 3 times per hand, with the final score being the maximum force exerted.

#### Cognitive battery

Cognitive tests were administered at the participants’ homes by experienced registered nurses. The cognitive battery of tests for the various domains was *Memory.* Verbal memory was assessed by a Prose Recall Test. This test is a Swedish language Prose Recall task similar to those from the Wechsler Memory Test (Wechsler, 1945). Respondents were asked for immediate free recall of a brief story.

Memory recognition and memory correspondence were assessed by four subtests of the Memory in Reality Test (Johansson, 1988/89): Naming (participant is shown and asked to name 10 objects), Recall (participant is asked to recall the 10 objects after a 30-min delay), Recognition (participant is shown objects not recalled and asked to name these), and Correspondence/Relocation (participant is asked to replace objects in their original locations). The maximum score for each subtest is 10 (Johansson, 1988/89).

Short-term memory was assessed by the Digit Span Forward and Backward Test (Wechsler Adult Intelligence Scale; Wechsler, 1991). The participants were asked to recall orally presented digits in the same and reverse order as they were presented. Higher scores represent higher levels of short-term memory.

##### Visuospatial ability and *reasoning*.

—Visuospatial ability was assessed with the Koh’s Block Design Test (3, from Dureman & Sälde, 1959). Respondents were asked to reproduce with blocks a pattern shown on cards. Higher scores are indicative of a greater level of visuospatial ability. Other tasks included a Clock drawing task from the Swedish Clock Test (Johansson & Zarit, 1991) and the Figure Logic reasoning test, which require participants to identify one figure out of five in a row that is different in concept from the rest (2, from Dureman & Sälde, 1959).

##### Motor and perceptual speed.

—A modified version of the speeded Digit–Symbol Substitution Test (Wechsler & De Lemos, 1981) was used which measures motor speed and accuracy. The participants were given a list of symbols associated with digits from 1 to 9 and instructed to orally fill in the blanks with the digit that corresponds to each symbol. The test score is the total number of correct sequential matching of digits to symbols in a 90-s interval. Another perceptual speed test, *Figure Identification*, Psif, from Dureman and Sälde (1959) was also used to assess perceptual speed. Participants are asked to match, as quickly as possible, a target figure with one identical figure placed in line among four others. The maximum score is 60 and the time limit is 4 min.

##### Crystallized abilities.

—These abilities can be described as the general knowledge acquired through education and other cultural experiences over the entire life span (Horn, 1991; Horn & Noll, 1997). In the OCTO-Twin Study, two tests from the Swedish version of the Wechsler Adult Intelligence Scale (Jonsson & Molander, 1964; Wechsler & De Lemos, 1981) were used: the Swedish version of the Information Task and the Synonyms Test. The Information Task includes questions of general knowledge, which requires participants to provide answers to questions assessing acquired semantic knowledge of facts. The Synonyms Test is a verbal meaning test where the respondent has to find a synonym that matches a target word among five alternatives. Higher scores represent higher levels of knowledge.

### Other Data

#### Sociodemographic data

Sociodemographic information included the participant’s sex, age at study entry, and years of education.

#### Body mass index

Body mass index (BMI) was derived as kg/m^2^ using height and weight measures collected by nurses.

#### Physical activity

Individuals were asked: “Are you presently doing, or have you previously done anything special to train your body or keep your body fit?” The possible responses were “no” (0), “yes, to some extent” (1), or “yes, to a great extent” (2) and responses coded on a scale from 0 to 2. Using these responses, we derived a binary indicator collapsing positive responses into a single category coded as 1 and negative responses into another category coded as 0.

### Analytical Approach

CD scores were derived using an adaptation of the method described by Holtzer et al. (2020, 2008). We only included data from individuals who were dementia-free at study entry. The method applies a z-transformation to the raw scores of each test using parameters from the distribution of the entire sample, and then, the application of the formula:


$$\begin{array}{l} Dispersion=\sqrt{\frac{{\sum_{k=1}^{k=K}\left(T_{ik}-S_{i}\right)}^{2}}{K-1}} \end{array}$$

where *T*_*ik*_ is the *k*th z-transformed test for participant *i*, *K* is the number of tests, and *S*_*i*_ is participant *i’*s mean of the transformed scores.

Average cognitive performance scores were derived by taking the mean of scores in the different cognitive tests.

To estimate grip strength trajectories, following evidence about sex differences in grip strength, we fitted a series of independent linear mixed-effects models to grip strength measures from men and women structured as a function of time in study. Initially, the intercept and slope parameters of the models were adjusted for CD, mean cognitive performance, age at study entry, and BMI (centered at their mean values). That is, CD for men was centered at 0.49 and for women at 0.53; mean cognitive function was centered at 0.11 and 0.08, respectively, for men and women, whereas BMI was centered at 24.4 and 24.9 for men and women, respectively, too. Physical activity was coded as 1 if the individuals trained their body and 0 if the individual did not train their body. We also included terms indicating whether individuals smoked or had smoked before entering the study (yes = 1, no = 0) and had a history of stroke (yes = 1, no = 0). Then, interaction terms between mean cognitive performance and dispersion were added to the models.

All models were estimated using Mplus, accounting for the correlations between twins using the clustering option available in the package. Models were estimated using maximum likelihood estimation, and missing data are assumed to be missing at random.

## Results

### Descriptive Statistics

**Figure 1.**
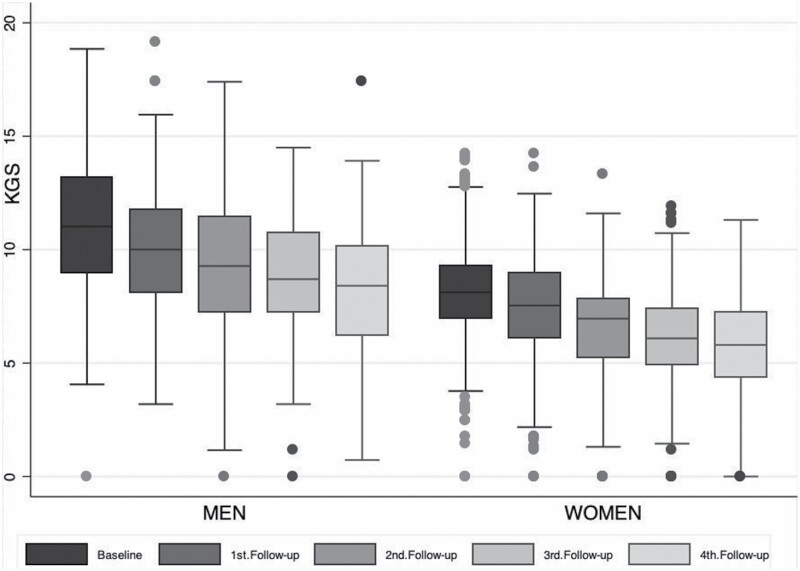
Box plots of mean cognitive scores and dispersion scores of men and women who were free from dementia at baseline.

The initial sample included 702 participants, of which there were 98 individuals who had been diagnosed with dementia at study entry. Of the 604 individuals who were free from dementia at baseline, there were 19 individuals (12 women, seven men) who had missing data in all cognitive tests included in the derivation of the dispersion index. Their grip strength baseline measures did not differ from the baseline grip strength measures of those who had valid cognitive scores (*t*-test (621) = 0.48, *p* < .05) and were, therefore, excluded from the analysis, and 12 individuals did not have data on education. Hence, the final analytical samples included 204 men and 373 women who were free from dementia at study entry. Women were older than men (83.58 [0.17] vs. 82.87 [SD = 0.18], *t*-test, *p* = .007) and had fewer years of education (7.02 [0.10] vs. 7.50 [0.20], *t*-test, *p* = .01). Yet, there were no statistically significant differences between men and women in BMI (24.42 [0.20] vs. 24.96 [0.25], *t*-test, *p* = .10), mean cognitive performance (0.11 [0.02] vs. 0.08 [0.04], *t*-test, *p* = .53), and CD (0.49 [0.02] vs. 0.53 [0.03], *t*-test, *p* = .08). See Figure 1 for box plots of mean cognitive performance and CD in the sample of men and women in the study and Table 1 for the descriptive characteristics of the results of each of the cognitive tests in men and women. In addition, there were differences between men and women in smoking habits (72% of men compared to 77% of women, χ ^2^ = 140.13, *p* = .001), engagement in physical activity (68.0% of men trained their body compared to 55.5% of women, χ ^2^ = 10.18, *p* = .006), but not in stroke prevalence before joining the study (χ ^2^ = 2.39, *p* = .30). Further demographic details are given in Table 2.

**Table 1.** Mean and Standard Deviation of Each of the Cognitive Tests Included in the Derivation of the Dispersion Scores in Men and Women

Cognitive test	Mean (*SD*)	
	Men	Women
Block design	11.36 (7.49)	11.70 (6.89)
Clock	13.84 (2.79)	13.85 (8.15)
Digit span backward	3.27 (1.49)	3.40 (1.47)
Digit span forward	5.62 (1.26)	5.39 (1.17)
Digit symbol	24.31 (10.85)	
Figure	15.57 (4.23)	15.45 (4.04)
Information	31.77 (9.16)	26.05 (11.52)
Recall	5.81 (2.42)	6.69 (2.39)
Correspondence	7.21 (2.44)	8.15 (2.21)
Recognition	9.89 (0.47)	9.61 (1.33)
Naming	9.89 (0.47)	10 (0.1)
Prose recall	9.13 (4.30)	9.90 (3.69)
Synonyms	16.64 (6.59)	16.92 (5.58)

**Table 2.** Descriptive Characteristics of the Sample

	Men (*N* = 204)	Women (*N* = 373)
	Mean (*SD*) or *n* (%)	Mean (*SD*) or *n* (%)
Baseline age	82.87 (2.67)	83.58 (3.20)
Education	7.50 (2.90)	7.02 (1.92)
Grip strength		
Baseline	11.06 (2.99)	8.11 (2.30)
First follow-up	9.99 (2.74)	7.43 (2.34)
Second follow-up	9.21 (3.28)	6.52 (2.40)
Third follow-up	8.80 (2.92)	6.09 (2.36)
Fourth follow-up	8.19 (3.21)	5.67 (2.22)
Body mass index	24.96 (3.37)	24.42 (3.84)
Mean cognitive function	0.08 (0.54)	0.11 (0.52)
Dispersion	0.53 (0.33)	0.49 (0.26)
Trains their body		
Yes	139 (68.1%)	207 (55.5%)
No	65 (31.9%)	162 (43.7%)
NA	0 (0.0%)	4 (1.0%)
Smoker		
Yes	147 (72.1%)	288 (77.2%)
No	57 (27.9%)	81 (21.7%)
NA	0 (0.0%)	4 (1.1%)
Stroke history		
Yes	23 (11.3%)	29 (7.8%)
No	179 (87.7%)	342 (92.0%)
NA	2 (1.0%)	2 (0.5%)

*Note: SD* = standard deviation; NA = not applicable.

#### Grip strength trajectories

**Figure 2.**
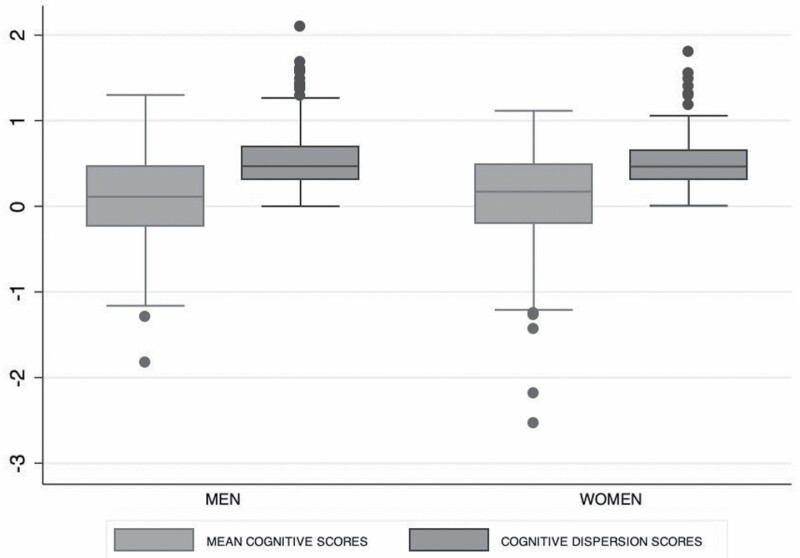
Box plots of grip strength measurements at baseline and at each of the study follow-up data collection waves for men and women who were free from dementia at study entry.

We present results from the longitudinal models fitted to the sample of men and women separately below. Box plots of grip strength measures at each study wave are presented in Figure 2.

#### Men’s grip strength trajectories

For a reference man (aged 83 years old at study entry, with an average BMI, and no history of stroke before entering the study, who trains his body, and has average CD and mean levels of cognition), grip strength at study entry was estimated at 111.57 (*SD* = 0.53) kg, with an annual rate of decline of 2.00 (*SD* = 0.33) kg, although this estimate did not reach conventional significance levels (see Table 3 for results).

**Table 3.** Results From the Linear Mixed Effects Model Fitted to Grip Strength Measures in a Sample of Participants in the OCTO-Twin Study Who Remained Free of Dementia During the Study Follow-Up

	Men		Women	
Fixed effects	Beta (*SE*) [95% CI]	*p*	Beta (*SE*) [95% CI]	*p*
*Grip strength level*	11.57 (0.53) [10.68, 12.44]	<.001	7.72 (0.32) [7.09, 8.34]	.000
Dispersion	−2.00 (0.73) [−3.46, −0.53]	.007	0.11 (0.51) [−0.20, −0.04]	.83
Mean cognitive function	2.18 (0.79) [0.71, 3.64]	.006	0.93 (0.51) [−0.05, 1.92]	.07
Baseline age	−0.19 (0.07) [−0.31, −0.06]	.01	−0.12 (0.05) [−0.20, −0.04]	.003
Body mass index	0.01 (0.05) [−0.11, 0.11]	.99	0.08 (0.03) [0.04, 0.13]	.002
Physically active	0.68 (0.42) [−0.07, 1.44]	.10	0.70 (0.28) [0.25, 1.14]	.002
Stroke	−0.59 (0.63) [−1.71, 0.53]	.35	−0.21 (0.42) [−1.02, 0.58]	.61
Dispersion × Mean cognitive performance	−3.16 (1.17) [−5.09, −1.20]	.007	−0.30 (0.86) [−1.75, 1.14]	.72
*Grip strength rate of change*	−0.45 (0.11) [−0.63, −0.26]	.00	−0.36 (0.02) [−0.47, −0.23]	.00
Dispersion	0.06 (0.15) [−0.24, 0.36]	.67	0.02 (0.09) [−0.15, 0.21]	.81
Mean cognitive function	−0.49 (0.15) [−0.87, −0.02]	.002	−0.002 (0.12) [−0.24, 0.24]	.99
Baseline age	−0.007 (0.02) [−0.03, 0.01]	.68	0.008 (0.005) [−0.01, 0.01]	.12
Body mass index	0.02 (0.01) [−0.03, 0.03]	.90	−0.008 (0.005) [−0.02, 0.15]	.12
Physically active	−0.06 (0.08) [−0.20, 0.07]	.45	−0.06 (0.04) [−0.13, 0.02]	.15
Stroke	−0.01 (0.14) [−0.32, 0.29]	.94	−0.15 (0.08) [−0.37, 0.06]	.04
Dispersion × Mean cognitive performance	1.00 (0.23) [0.41, 1.57]	.00	0.17 (0.16) [−0.25, 0.60]	.29

*Notes:* CI = confidence interval; *SE* = standard error. Random effects: intercept 2.13, rate of change 0.15, error 1.21, corr (intercept, slope) = −0.49.

**Figure 3.**
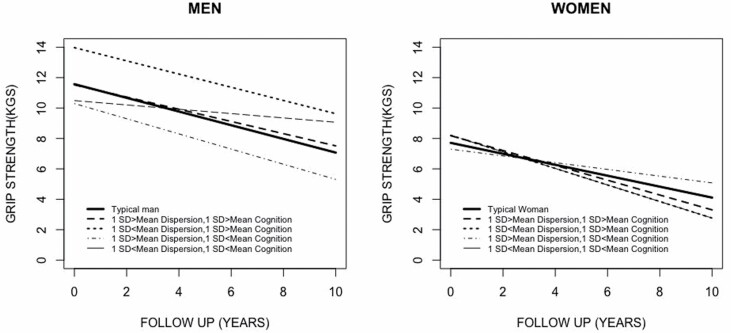
Estimated trajectories of a typical man and woman and for individuals with various levels of mean and dispersion scores. *Notes:* Sample sizes for groups were as follows: 1 *SD* > mean dispersion, 1 *SD* > mean cognition: *n* = 30; 1 *SD* < mean dispersion, 1 *SD* > mean cognition: *n* = 26; 1 *SD* > mean dispersion, 1 *SD* < mean dispersion: *n* = 113; 1 *SD* < mean dispersion, 1 *SD* < mean cog: *n* = 35.

Results suggest that the association of CD scores with baseline grip strength and its annual rate of decline vary with the level of mean cognitive function. We found that for the same level of average cognitive function, men with more dispersed scores showed weaker grip and they declined at a slower rate than men with less dispersed cognitive scores. See Figure 3 for a graphical representation of grip strength trajectories for prototypical individuals.

Except for older baseline age that had a negative association with grip strength at study entry, no other factor emerged as associated with grip trajectories.

#### Women’s grip strength trajectories

For a reference woman, grip strength at study entry was estimated at 7.72 (*SD* = 0.32) kg with a rate of decline of −0.36 (*SD* = 0.02) kg/year. Contrary to results in the sample of men, neither CD nor mean cognitive performance emerged as associated with baseline grip strength level or its rate of decline. Older baseline age (−0.12 [*SD* = 0.05]) was associated with weaker grip strength, whereas higher BMI and physical activity were associated with stronger grip (0.08 [*SD* = 0.03] and 0.70 [*SD* = 0.28], respectively). Finally, women who had a history of stroke declined at a faster rate than unaffected women (−0.16 [*SD* = 0.08]).

## Discussion

In our investigation exploring whether dispersion in cognitive function is associated with grip strength trajectories in a sample of the oldest old, we found that the association varied between men and women. While in men we found an association that varied by level of mean cognitive function, no association was found between CD or cognitive mean function in the sample of women. Hence, our hypotheses that CD would be negatively associated with grip strength level and positively associated with the rate of change (i.e., decline) in men *and* women were only partially confirmed by our findings.

The question of whether cognitive function is associated with physical function, and in particular with grip strength, an indicator of biological vitality, has been studied extensively following the postulation of the common-cause hypothesis (Baltes & Lindenberger, 1997), whereby it has been suggested that cognition and grip strength share similar variability of change that is influenced by a common neurological cause (Bjork et al., 2016). The relationship between CD and grip strength can be similarly explained, but perhaps CD, relative to mean level cognitive function, captures more insidious brain aging changes (Bangen et al., 2019). To date, most investigations have focused on associations between mean levels of specific cognitive domains and grip strength in cross-sectional and longitudinal studies. Although previous studies have linked CD with deteriorating trajectories of functional indicators (Bangen et al., 2019; Fellows & Schmitter-Edgecombe, 2015), to the best of our knowledge, our study is the first that specifically examined the association of CD with grip strength.

Reports of the associations between CD and poorer white matter integrity (Halliday et al., 2018) and with some but not all cerebrospinal fluid biomarkers of Alzheimer’s disease (Duchek et al., 2009; Gleason et al., 2018; Watermeyer et al., 2020b) have led researchers to postulate dispersion as an early marker of pathological brain changes. Yet, as these investigations were conducted in samples of younger adults, the question about the onset and timing of the association of dispersion with different markers of deterioration remains to be further investigated. Previous work with the OCTO-Twin cohort found that rates of decline in cognitive function were closely related to the rate of decline in grip strength before death (Björk et al., 2016), supporting the terminal decline hypothesis, in which proximity to death is accompanied by a sharper cognitive decline (Muniz-Terrera et al., 2011). Our study with the same cohort differs from this report in that we focused on grip strength changes outside the context of proximate death and we were interested in CD, not level of cognitive performance, as a marker for grip decline, and thus, age-related change. Relative to the level of cognitive performance, increased dispersion may reflect more subtle disintegration and/or reorganization of neural processes and activities in aging individuals before the onset of overt cognitive symptoms are detectable through traditional methods (Holtzer et al., 2020). Therefore, incorporation of this measure alongside the average level of cognitive performance might offer earlier adjunct information regarding an individual’s neurocognitive integrity and future decline in vitality, as measured by grip strength.

 Indeed, in a previous report where we studied the association of dispersion with dementia risk in a sample of OCTO-Twin participants, we found that individuals with greater CD scores have an increased risk of dementia, above and beyond controlling for level of cognitive performance (Watermeyer et al., 2020a). This previous investigation, taken together with our new analysis of grip strength trajectories, partly supports the common-cause hypothesis although some questions remain unanswered.

We only found an association between CD and grip strength trajectories in the sample of men but not women, despite no significant differences in dispersion or mean cognitive functioning between men and women. Although women were slightly older than men, their mean and dispersion cognitive scores were not significantly different. Instead, within the female sample, BMI and being physically active correlated with grip strength at baseline, while only a previous incident of stroke related to the rate of change in grip strength. Such gender differences add to the small but growing literature showing gender-specific predictors of physical status in mid-to-later life (Fragala et al., 2012; Mohd Hairi et al., 2010) and the oldest old (Parker et al., 1996) and support the call for integration of sex and gender in health promotion and intervention research (Mauvais-Jarvis et al., 2020; Tannenbaum et al., 2016). Furthermore, although the examination of incident dementia in the sample of men and women showed that there were fewer men than women (48 vs. 79) who received a diagnosis of dementia during the study follow-up, their age at diagnosis was only borderline significantly different (m: 86.04 [0.49] vs. f: 87.29 [0.21], *t*-test, *p* = .005) and there were significant differences in the time past between diagnosis and study entry (m: 3.61 [0.34] vs. f: 3.76 [0.29], *t*-test, *p* = .74). Hence, it is unlikely that the differences in our results are explained by proximity to dementia diagnosis. We therefore encourage further investigations to fully understand these results.

Our research has some limitations. To begin with, we assumed missing data are missing at random, an assumption that may not be fulfilled. Because of the relatively small number of individuals in the subsample of men and women, we only included a core set of variables in our analysis. For example, we did not account for comorbidities such as diabetes or many healthy lifestyle variables, only controlling for a simple measure of physical activity. This study was initiated already in 1991, when research about the role of lifestyle on the preservation of function was still in early stages and, therefore, the physical activity question asked is not optimal judging by current standards. Similarly, the construction of our dispersion metric is restricted by the tests available from the neuropsychological battery. The domains covered by the 15 tests used in this study do not have sufficient overlap to derive within-domain dispersion scores. Instead, our dispersion composite captures multiple domains of cognitive functioning and thus for any individual participant could reflect, for example, high inconsistent performance within a single cognitive domain or milder variability across multiple domains. This might minimize the clinical significance of our findings. Nonetheless, using the same construction approach, previous work has demonstrated that variability across tests was sensitive to dementia diagnosis, above and beyond average cognitive performance, suggesting our metric may possess incremental validity in predicting cognitive decline (Holtzer et al., 2008; Watermeyer et al., 2020a). Furthermore, performance variability across tests correlates poorly with global cognitive indices (Watermeyer et al., 2020a), suggesting that such scores might reflect distinct aspects of brain health and/or cognitive aging. Future work is required to determine the best construction method to optimize sensitivity and specificity for physical decline, neuropathology, and cognitive decline to support the dispersion metric’s clinical relevance.

Notwithstanding these limitations, our study’s strengths lie in its longitudinal design and relatively well-characterized oldest-old cohort. Furthermore, this is the first investigation of the association of CD with grip strength, a marker of biological vitality or aging. This work opens several lines of future research: We strongly encourage replication of our methods in other samples of older adults and further examination of whether the timing of associations between CD and grip strength differs by sex.
